# Olive Cultivation in the Southern Hemisphere: Flowering, Water Requirements and Oil Quality Responses to New Crop Environments

**DOI:** 10.3389/fpls.2017.01830

**Published:** 2017-10-27

**Authors:** Mariela Torres, Pierluigi Pierantozzi, Peter Searles, M. Cecilia Rousseaux, Georgina García-Inza, Andrea Miserere, Romina Bodoira, Cibeles Contreras, Damián Maestri

**Affiliations:** ^1^Estación Experimental Agropecuaria San Juan, Instituto Nacional de Tecnología Agropecuaria (Inta), CONICET, San Juan, Argentina; ^2^Centro Regional de Investigaciones Científicas y Transferencia Tecnológica de La Rioja (CRILAR, Provincia de La Rioja, UNLaR, SEGEMAR, UNCa, CONICET), La Rioja, Argentina; ^3^Instituto Multidisciplinario de Biología Vegetal, Consejo Nacional de Investigaciones Científicas y Técnicas, Universidad Nacional de Córdoba, Córdoba, Argentina

**Keywords:** chilling requirements, fatty acids, irrigation, oil concentration, oil yield, water requirements, *Olea europaea* L.

## Abstract

Olive (*Olea europaea* L.) is a crop well adapted to the environmental conditions prevailing in the Mediterranean Basin. Nevertheless, the increasing international demand for olive oil and table olives in the last two decades has led to expansion of olive cultivation in some countries of the southern hemisphere, notably in Argentina, Chile, Perú and Australia. While the percentage of world production represented by these countries is still low, many of the new production regions do not have typical Mediterranean climates, and some are located at subtropical latitudes where there is relatively little information about crop function. Thus, the primary objective of this review was to assess recently published scientific literature on olive cultivation in these new crop environments. The review focuses on three main aspects: (a) chilling requirements for flowering, (b) water requirements and irrigation management, and (c) environmental effects on fruit oil concentration and quality. In many arid and semiarid regions of South America, temperatures are high and rainfall is low in the winter and early spring months compared to conditions in much of the Mediterranean Basin. High temperatures have often been found to have detrimental effects on olive flowering in many olive cultivars that have been introduced to South America, and a better understanding of chilling requirements is needed. Lack of rainfall in the winter and spring also has resulted in an urgent need to evaluate water requirements from the flower differentiation period in the winter to early fruit bearing. Additionally, in some olive growing areas of South America and Australia, high early season temperatures affect the timing of phenological events such that the onset of oil synthesis occurs sooner than in the Mediterranean Basin with most oil accumulation taking place in the summer when temperatures are very high. Increasing mean daily temperatures have been demonstrated to decrease fruit oil concentration (%) and negatively affect some aspects of oil quality based on both correlative field studies and manipulative experiments. From a practical standpoint, current findings could be used as approximate tools to determine whether the temperature conditions in a proposed new growing region are appropriate for achieving sustainable oil productivity and quality.

## Introduction

The geographic origin of cultivated olive (*Olea europaea* L.) can be traced to areas along the eastern Mediterranean coast where Turkey, Syria, Lebanon, Palestine, and Israel are currently located. Some records indicate that olive trees have been cultivated in those areas since at least 3000 BC (Connor, 2005). Olive then spread widely around southern Europe, northern Africa, and the Iberian Peninsula. Today, approximately 98% of olives are cultivated in Mediterranean Basin countries. Spain, Italy, and Greece together produce about 77% of the world's olive oil. Portugal, Tunisia, Turkey, Morocco, Syria, and Egypt also have an important amount of production, but oil yields are low per hectare in many instances and modern processing technology is underutilized (El-Kholy, 2012).

In the last 20–30 years, interest in olive oil production and consumption has expanded olive cultivation to regions and countries outside the Mediterranean Basin such as Australia, China, India, and South America. In the southern hemisphere, the biggest hectarage for olive cultivation is located in Argentina. Until 1990, olive cultivation covered a total area of approximately 30,000 ha, most of which corresponded to small orchards (<10 ha) with traditional management (i.e., low planting density and flood irrigation). Subsequent tax exemption laws brought in large investments that included large commercial orchards (>100 ha) with higher planting densities and drip irrigation. Currently, there are about 110,000 ha under cultivation mainly in the central-western and north-western regions bordering the Andes mountain range (27–33°S latitude). Spanish and Italian cultivars including “Arbequina,” “Manzanilla,” “Picual,” and “Frantoio” have been extensively planted with about 70% of production being devoted to olive oil. “Arauco” is the only cultivar recognized from Argentina in the World Catalog of Olive Varieties (IOOC, 2000).

In South America, Chile is ranked second in area planted with about 24,000 ha of olive orchards. Production is almost exclusively dedicated to olive oil, and similarly to Argentina, the most important cultivars are Spanish and Italian cultivars with “Arbequina” comprising 50% of the total production area. Other cultivars include “Frantoio,” “Arbosana,” “Picual,” and “Leccino.” “Azapa” is a local table olive cultivar with a close resemblance to “Arauco.” Perú (approximately 28,000 ha of olive orchards) is not a large producer of olive oil, but table olive production has increased considerably over the last decade. Being located near the equator, the climate conditions in Perú are very different from those found in traditional olive growing regions (Ayerza and Sibbett, 2001). Uruguay and Brazil both have minor, but increasing olive production areas (10,000 and 1,300 ha, respectively), as a result of ongoing expansion projects that are mainly for oil production.

Currently, Australia has about 11 million olive trees spread across approximately 35,000 ha. Although early orchards included a large number of cultivars, about 90% of Australian olive oil is produced from common European cultivars (“Arbequina,” “Frantoio,” “Coratina,” “Corregiola,” “Manzanilla,” “Picual,” and “Koroneiki”) and more recently from the Israeli cv. Barnea. Australian areas under olive cultivation include a wide natural diversity of environments from the most southern point of Western Australia to the northern tropical areas of Queensland. Olive production has expanded rapidly in recent years due to the adaptation of intensive and super-high density planting systems in new commercial orchards. As a result of the low rainfall and the unpredictable nature of Australian olive crop environments, almost all Australian olive orchards are irrigated (Mailer, 2012). This is also the case in Argentina because annual rainfall is most often between 100 and 400 mm (Searles et al., 2011).

At this point, it is important to bear in mind that many of the olive growing areas in the southern hemisphere have temperature and precipitation regimes that are very different from those of the Mediterranean Basin where olive trees are traditionally cultivated (Table 1). This reality has encouraged, or even forced, both growers and academics to seek new approaches to crop management. Although scientific studies conducted outside the Mediterranean Basin are still limited, it is important to review and synthesize the knowledge currently available on several critical topics. Lavee (2014) provided some general guidelines on olive adaptation to new environments based mostly on knowledge from the Mediterranean Basin, and concluded that there is a strong need for local research in new production areas. For these reasons, the primary objective of this review was to analyze recently published scientific literature on olive cultivation in non-Mediterranean environments in the southern hemisphere. The review focuses on three main aspects: (a) chilling requirements for flowering, (b) water requirements and irrigation management, and (c) environmental effects on fruit oil concentration and quality. The revision also contributes to identifying areas where knowledge is insufficient and to set priorities for further research.

**Table 1:** Temperature, rainfall, and evapotranspiration (ETo) values from different olive growing areas in South America and Australia compared with those of typical Mediterranean regions in Spain, Italy, and Tunisia.

**Location**	**Parameter**	**Spring**	**Summer**	**Autumn**	**Winter**	**Annual**
Argentina (North-western region)	Tmax	29.1	33.5	26.3	20.1	27.2
Latitude 27°-29°S	Tmin	13.8	19.5	13	4.2	12.6
Altitude (masl) 420–1,200	Rainfall (mm)	48.5	166	73.0	10.0	298
	ETo (mm)	483	555	339	243	1,620
Argentina (Central región)	Tmax	27.5	33.8	25.3	20.9	26.1
Latitude 30.5°S	Tmin	10.6	18.1	10.9	6.35	10.3
Altitude (masl) 450	Rainfall (mm)	92.4	330	120	10.0	556
	ETo (mm)	405	556	230	178	1,369
Argentina (Central-western region)	Tmax	28.5	32.0	21.2	18.2	25.3
Latitude 31°-33°S	Tmin	13.8	18.5	7.5	2.8	11.0
Altitude (masl) 590–920	Rainfall (mm)	38.5	73.9	18.6	9.8	141
	ETo (mm)	484.	538.	216.	196	1,435
Peru (South-western Coast,	Tmax	23.4	28.1	22.7	17.8	23.0
e.g., Tacna and Ilo valleys)	Tmin	13.5	18.1	13.7	10.0	13.8
Latitude 17°-18°S	Rainfall (mm)	3	7.3	1.4	14.5	26.2
Altitude (masl) 895	ETo (mm)	434	491	278	226	1,429
Brazil (South-eastern region,	Tmax	27.7	27.6	23.2	23.8	25.6
Minas Gerais state)	Tmin	14.8	14.9	10.6	9.2	12.4
Latitude 22°-23°S	Rainfall (mm)	376	492	140	247	1,255
Altitude (masl) 1,200–1,300	ETo (mm)	343	373	233	212	1,159
Uruguay (South-eastern region,	Tmax	23.3	27.9	20.8	17.1	22.3
e.g., Rocha)	Tmin	11.8	15.7	9.6	7.0	11.0
Latitude 33°-33.6 S	Rainfall (mm)	279	316	271	342	1,208
Altitude (masl) 4–63	ETo (mm)	NA	NA	NA	NA	NA
Australia (South-eastern region,	Tmax	25.7	30.5	20.6	16.1	23.3
e.g., Victoria, New South Wales)	Tmin	11.0	15.5	7.7	4.1	9.6
Latitude 31°-36°S	Rainfall (mm)	179	170	163	183	695
Altitude (masl) 150–420	ETo (mm)	426	501	219	192	1,338
Australia (South-western region,	Tmax	23.4	29.2	22.3	17.8	23.2
e.g., Perth)	Tmin	11.3	15.1	10.4	7.7	11.1
Latitude 30°-33°S	Rainfall (mm)	93.6	33.0	238	358	723
Altitude (masl) 5	ETo (mm)	392	467	228	189	1,276
Spain (Southern region, e.g., Sevilla)	Tmax	23.2	34.0	26.0	17.1	25.1
Latitude 37.2°-37.6°N	Tmin	10.6	18.3	13.5	6.60	12.2
Altitude (masl) 8–358	Rainfall (mm)	134	20	167	233	554
	ETo (mm)	372	600	288	147	1,408
Spain (Central region, e.g., Toledo)	Tmax	19.7	31.9	21.7	12.1	21.3
Latitude 39°-39.5° N	Tmin	7.5	17.3	10.0	2.5	9.3
Altitude (masl) 510	Rainfall (mm)	110	49	100	100	359
	ETo (mm)	324	556	238	107	1,225
Italy (Southern region, e.g., Benevento)	Tmax	19.7	22.5	14.4	8.0	16.2
Latitude 41.1° N	Tmin	10.8	18.2	6.1	5.0	10.0
Altitude (masl) 250	Rainfall (mm)	153	123	263	189	728
	ETo (mm)	432	546	125	129	1,232
Tunisia (Eastern coast, e.g., Sousse)	Tmax	19.7	31.9	21.7	12.1	21.3
Latitude 39°-39.5° N	Tmin	7.5	17.3	10.0	2.5	9.3
Altitude (masl) 516	Rainfall (mm)	110	49	100	100	359
	ETo (mm)	324	556	238	107	1,225

*Tmax and Tmin are the average seasonal maximum and minimum temperatures (°C), respectively. NA, not available. ETo values were calculated using the FAO Penman-Monteith method (Allen et al., 1998)*.

### Chilling requirements for flowering

Olive is a crop that flowers profusely and produces high olive oil yields under the prevailing climatic and agro-ecological conditions of the Mediterranean Basin with most production being confined traditionally to latitudes between 30° and 45° North. Yet, olive trees have the ability to adjust to a wide range of different environments due to a number of specific biological and anatomical characteristics (Gucci and Caruso, 2011). This adaptation often leads to significant effects on several aspects of reproductive performance such as flowering, oil yield, and oil quality that vary depending on the environmental conditions (e.g., Tura et al., 2007; Lazzez et al., 2008; Temine et al., 2008; Torres et al., 2009; Di Vaio et al., 2012; Rondanini et al., 2014). Rapoport (2014) has reviewed many of the reproductive biology responses to drought and temperature in olive under extreme conditions. Thus, our aim in this section is to focus on the specific issue of chilling hours for flowering, which is of great importance in many production areas in the southern hemisphere.

Flowering is one of the major yield determinants in olive, and although olive trees are capable of producing a large number of flowers, the percentage of flowers that set fruit is usually very low with values of about 2% (Lavee et al., 1996). Flowering occurs once buds induced the previous growing season receive sufficient chilling during the winter dormancy period to end dormancy, differentiate anatomically, and accumulate warmer temperatures adequate for budburst (Rallo and Cuevas, 2008). The accumulation of chilling requirements for flowering during winter dormancy is most often referred to as vernalization, and high temperatures during the winter may adversely affect the number of chilling hours accrued (Malik and Perez, 2011). Thus, flowering and therefore fruiting may be reduced due to insufficient chilling temperatures at low latitudes (<30°).

**Figure 1:**
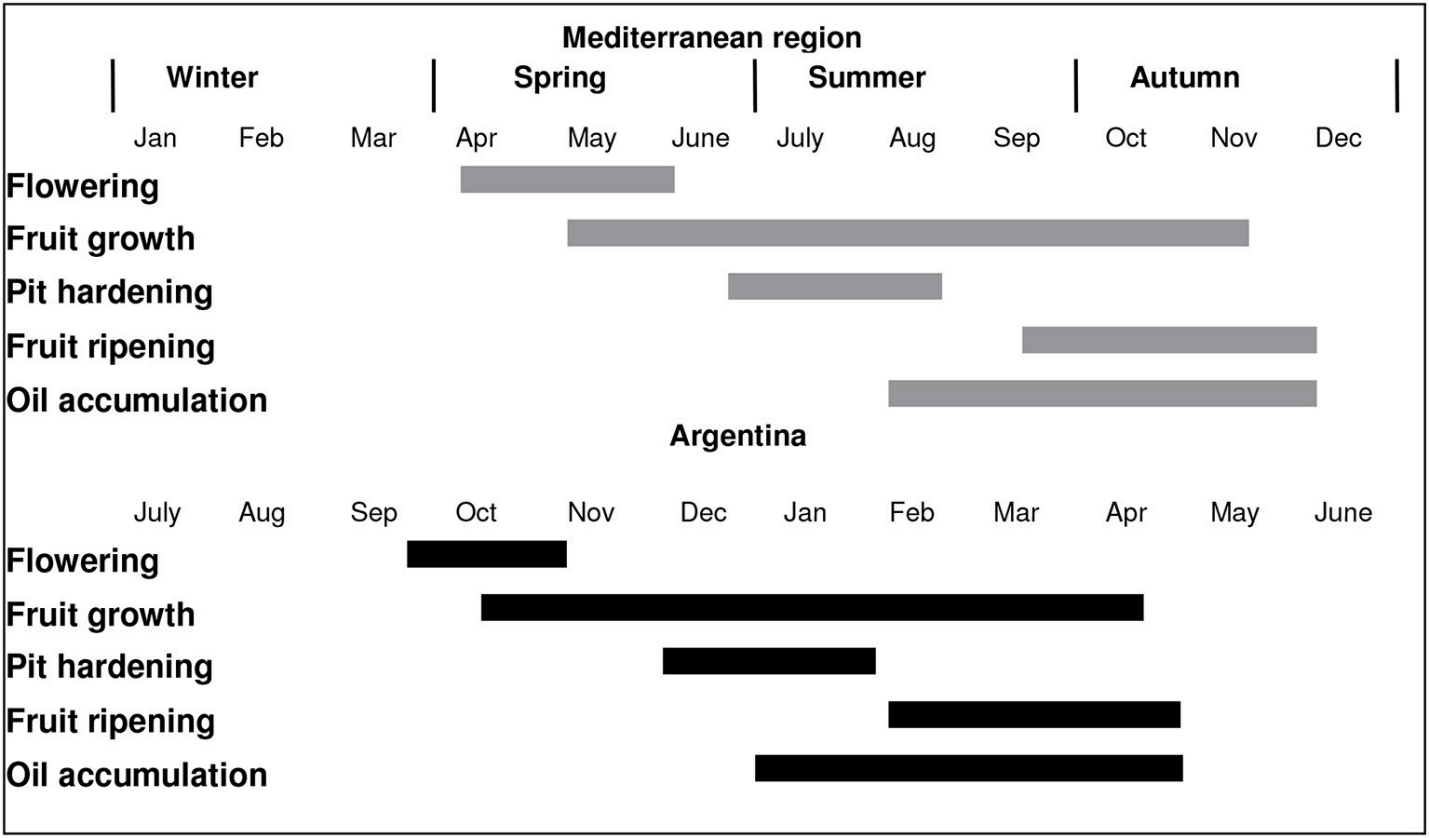
The chronological sequence of the main phenological stages of olive cultivation in the Mediterranean Basin compared with the main olive growing regions in Argentina. The Mediterranean and Argentine sequences are adapted from Sanz-Cortés et al. (2002) and Gómez del Campo et al. (2010), respectively.

**Figure 2:**
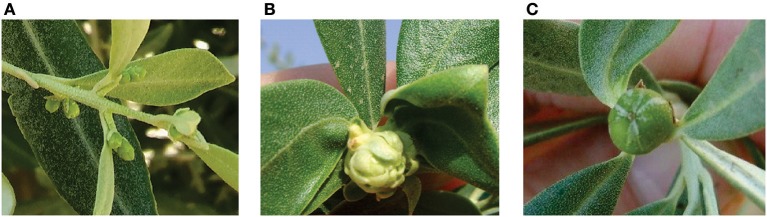
Deformed floral buds and fruit from olive trees exposed to insufficient chilling temperatures and high temperature events. Photograph **(A)** is from the olive collection at INTA-San Juan (31°S, Argentina); photographs **(B,C)** are from semi-tropical regions of Brazil, 22°-23°S (courtesy of Dr. Shimon Lavee).

Figure 1 shows the chronological sequence of the main phenological stages of olive cultivation in the Mediterranean Basin compared with the main growing regions in Argentina (i.e., central-western and north-western Argentina). In NW Argentina, it has been observed that fairly high winter and spring temperatures lead to earlier flowering, and eventually to earlier oil accumulation, relative to the Mediterranean Basin (Gómez del Campo et al., 2010). Early flowering was also reported in other low latitude South American production areas such as Perú (Lavee, 2014). High temperatures have been shown to result in a lack of chilling hours for flowering in some cultivars growing in NW Argentina (Aybar et al., 2015) and in Tacna, Perú (Castillo-Llanque et al., 2014). In addition, it has been observed that trees exposed to insufficient chilling temperatures and high temperature events can flower, but the flowers are of low quality and have a low set percentage. This phenomenon has been documented in olive growing areas at low latitudes where some olive varieties produce deformed floral buds and fruit (Figure 2). This is in accordance with previous suggestions that winter chilling is necessary not only for floral differentiation, but also for proper formation of floral buds (Rallo and Martin, 1991; De Melo-Abreu et al., 2004).

The optimum temperature regime for reproductive development of olive buds has been considered to include fluctuating temperatures from 2 to 19°C (Denney and McEachern, 1983). However, in greenhouse experiments under controlled temperature conditions, Malik and Bradford (2009) observed maximal flowering in “Arbequina” at minimum temperatures ranging from 4.4 to 7.8°C and flowering reductions when night temperature was maintained at 2.2°C. Hence, these authors suggest an inhibitory effect on flowering even at temperatures that were previously considered satisfactory for reaching chilling requirements.

At a large geographical scale, the potential of new sites for olive cultivation in the Arid Chaco Region in northern Argentina was assessed through temperature regime comparisons with more established sites in central Argentina, Italy, Spain, and the USA (Ayerza and Sibbett, 2001). At these sites, the frequencies of minimum (0.0–12.5°C) and maximum (12.5–21.1°C) temperatures during the winter were determined along with those of extreme cold (<0°C) and heat (>37°C) during the flowering period. Following these criteria, all Italian and Spanish olive growing sites had at least 150 days per year that were adequate for chilling hour accumulation, while some established Argentinean sites (San Juan, 31° 34' S; Mendoza, 32° 50' S) did not exceed 110 days, and all proposed new Arid Chaco sites had less than 60 days. Another distinctive feature of the climate in the Arid Chaco region during the winter season when chilling accumulation occurs is alternating periods (i.e., several days) of high and low temperatures (Ayerza and Sibbett, 2001). This reality may add an additional drawback for flower development and fruit set because temperatures above 21°C partially reverse chilling accumulation when they occur before chilling requirements are completed (De Melo-Abreu et al., 2004; Malik and Perez, 2011).

Lavee (2014) observed that the cold Humboldt Current along the Pacific coast of Perú lowers the expected temperatures for this latitude (<20°S). Interestingly, both an introduced Italian cultivar (“Frantoio”) and a local Peruvian cultivar (“Criolla”) clearly flower, in spite of the latitude. Castillo-Llanque et al. (2014) reported that this latter cultivar shows greater and more rapid floral bud development suggesting that it may be better adapted than the Italian cultivar to the local climatic conditions. Reductions in light intensity during the winter months due to continuous cloudiness in coastal Perú have also been suggested anecdotally to compensate for insufficient chilling (Lavee, 2014).

Even from casual observations, it seems clear that olive cultivars differ in their chilling requirement. An analytical approach for assessing the potential for flowering occurrence and date in different cultivars involves simulation models based on cultivar-specific thermal requirements (De Melo-Abreu et al., 2004). One of the models proposed by De Melo-Abreu et al. (2004) predicts the date when the chilling requirement is reached as well as the full flowering date after the accumulation of warm temperatures. Recently, this model was validated over several years at eight low latitude sites in north-western Argentina (Aybar et al., 2015). In “Arbequina,” normal flowering was observed at almost all sites and in all years, while normal flowering events in “Frantoio” and “Leccino” were uncommon. The results confirmed that these two latter cultivars require a very high number of chilling units in accordance with values from the World Olive Germplasm Bank of Córdoba (Spain), and winter temperatures in NW Argentina do not meet their chilling requirements for normal flowering in most years. In several tropical areas, expansion of fruit trees has been made possible by the use of growth regulators, which replace chilling requirements to overcome endodormancy. For example, hydrogen cyanamide (HC) has been used successfully in apple trees (Mohamed, 2008), grapes (Or et al., 2000), and peaches (Marodin et al., 2002). Benzyladenine (BA), a synthetic cytokinin that acts during dormancy release in apple (Bubán, 2000), is another growth regulator often used. However, BA did not affect flower initiation in the olive cv. Sevillano (Badr and Hartmann, 1972), and a more recent field trial in NW Argentina did not successfully lead to normal flowering in “Frantoio” when BA or HC were applied (Aybar, 2010).

From this section, we conclude that insufficient chilling hours during winter dormancy in many areas of South America and potentially other parts of the southern hemisphere often lead to reductions in flowering in some cultivars. Thus, cultivar-specific simulation models are recommended as approximate tools to predict whether individual cultivars will likely flower in proposed new growing regions.

### Water requirements and irrigation management

Olive has been cultivated traditionally under rainfed conditions in the Mediterranean Basin without supplemental irrigation (Connor and Fereres, 2005). Good olive oil yields can be obtained there without irrigation in growing areas where annual rainfall is greater than 600 mm (Gucci and Fereres, 2012). Although such a value may serve as a benchmark for olive cultivation in many Mediterranean countries, additional factors such as rainfall seasonality, soil water-holding capacity, and crop evapotranspiration should be carefully considered for specific growing regions.

Since the early pioneering study of Hartmann and Panetsos (1961) in California, several dozen studies have addressed the irrigation needs of olive trees growing under Mediterranean climate conditions based on the sensibility of the main olive phenological stages to water deficit (e.g., Tognetti et al., 2005; Lavee et al., 2007; Gucci et al., 2009; Rapoport et al., 2012; Gómez-del-Campo et al., 2014). These studies have mostly focused on evaluating irrigation needs under the hot dry summer conditions when rainfall is limited and provide the physiological and agronomic knowledge necessary for the application of irrigation strategies under many commercial growing conditions (Fernández, 2014). However, some recent advances in our understanding of olive tree response to irrigation have been possible due to the different climate regimes found in South America and elsewhere in the southern hemisphere.

In the Mediterranean Basin, irrigation is normally suspended during the winter months because rainfall is more than sufficient to satisfy crop evapotranspiration (ETc) under the fairly cold and cloudy conditions. The soil moisture stored during the winter also may preclude the need to irrigate in the spring during flowering and subsequent fruit set. By contrast, in many southern hemisphere climates where olive is cultivated (e.g., the subtropics of Australia and Argentina), rainfall events occur mostly in the summer with little or no winter rainfall. Somewhat greater temperatures during the winter and spring months at these latitudes compared to those of the Mediterranean also suggest that ETc should be higher. Thus, there are not irrigation experiences from Mediterranean countries that are applicable to these regions for this time of the year.

In the last several years, some studies evaluating irrigation needs during the winter have been conducted in the arid and semi-arid regions of central and North-western Argentina. A preliminary study by Rousseaux et al. (2008) examined some physiological and yield responses to the suspension of irrigation for 6–7 weeks during the winter season (July-August) in La Rioja province (NW Argentina). Soil water content decreased after 15 days in non-irrigated plots, and both pre-dawn and midday leaf water potential showed mild reductions during most of the experiment. However, this change in leaf water status was not enough to significantly decrease net photosynthetic rate, and fruit yield at harvest the following growing season only showed a modest percentage reduction (−20%). Estimates from this study suggested that little irrigation would be needed to satisfy crop water demand during the winter. Later sap flow measurements in whole trees combined with soil micro-lysimeter data during the winter also confirmed that ETc was only about 40% of reference transpiration (*sensu* Allen et al., 1998). Although maximum daily temperature was often around 20°C, transpiration per unit leaf area was minimal when daily average temperature was below 13°C (Rousseaux et al., 2009), a condition frequently observed during July-August due to low night temperatures.

In another study (Pierantozzi et al., 2013), when the deficit irrigation period was extended from early winter until mid-spring (i.e., mid-June through October), significant reductions in both photosynthetic pigment levels and net photosynthetic rates were observed in trees grown under moderate (50% ETc) and severe (25 and 0% ETc) deficit irrigation. Vegetative shoot growth started toward the end of the winter, and was significantly reduced by deficit irrigation (Pierantozzi et al., 2014). Flowering was also delayed in the trees receiving no irrigation. This delay, as well as reductions in flowering intensity, ultimately decreased oil yield in the treatments under moderate or severe water stress compared to the well-irrigated treatments (100 or 75% ETc).

The greater responses to deficit irrigation in Pierantozzi et al. (2013, 2014) than in Rousseaux et al. (2008) are most likely explained by the duration of the deficit period. When the influence of water availability was partitioned in four periods, water deficit during winter dormancy did not affect either flowering or fruiting parameters, but deficit during inflorescence development reduced many different flowering traits and ovule development (Rapoport et al., 2012). Thus, it appears that a short and mild water deficit during the colder, winter dormancy period (Rousseaux et al., 2008) may not greatly affect reproductive responses, but intensifying water deficit from late fall through mid-spring including winter dormancy, flower differentiation, and flower opening leads to detrimental effects (Pierantozzi et al., 2013, 2014). According to these two latter studies, little irrigation (50% ETc) may be sufficient to maintain adequate plant water potentials for the coldest winter months, but high (75% ETc) or full (100% ETc) irrigation rates could be needed by mid-August, which is approximately 2 months before full flowering, to guarantee adequate oil yields in regions with dry winter and spring seasons.

Many South American olive cultivation regions in Argentina, Chile and Perú are located at subtropical latitudes (<30°S) where high temperatures will likely affect annual water requirements. In a field experiment in NW Argentina (28° 33' S, province of La Rioja, Argentina), the warm climate facilitated excessive shoot growth when very high irrigation levels were applied (Correa-Tedesco et al., 2010). Based on both vegetative growth and fruit yield, an optimal crop coefficient (Kc) value of approximately 0.70 was determined for the entire growing season in this drip-irrigated, intensively-planted orchard (“Manzanilla fina”). According to these authors, such a Kc value may be sufficient to maintain the vegetative growth at an appropriate level without adversely affecting fruit yield. Based on transpiration and soil evaporation values in the same orchard, Rousseaux et al. (2009) calculated Kc values of about 0.65–0.70 and 0.85–0.90 for either moderately or excessively irrigated olive plots, respectively. The proposed Kc value of 0.7 is similar to reported values for many areas with Mediterranean climates such as California (Goldhamer et al., 1994) and Spain (Girona et al., 2002). However, the reference ET values in NW Argentina (1,600 mm) are higher than in most of the Mediterranean Basin (1,000–1,400 mm). These high ET values require a greater amount of total annual water in order to satisfy crop demand. Additionally, due to the low annual rainfall (100–400 mm) in this region, a very large proportion of the total water applied must be in the form of irrigation, rather than relying on rainfall.

Irrigation studies from Australia also provide partial information about olive water requirements and irrigation management (Yunusa et al., 2008; Zeleke et al., 2012; Zeleke, 2014). Estimates of soil water use from neutron probe measurements and canopy transpiration from porometer readings were obtained in four olive orchards in southern Australia (34°S) by Yunusa et al. (2008). Similar to NW Argentina, these orchards experienced high annual potential ET conditions (1,600 mm) with little rainfall during much of the growing season. It was observed that crop evapotranspiration in these orchards was fairly low (600 mm; Kc = 0.4) primarily because they were not irrigated during the spring when rainfall was scarce. Based on the results of Pierantozzi et al. (2013, 2014) from Argentina, yield in these orchards might be significantly increased by irrigating during the spring when flowering, fruit set, and most shoot growth occur. In an area with a more evenly distributed rainfall regime, a water balance approach indicated that annual ETc was about 700 mm, but a yearly value for reference evapotranspiration was not given (Zeleke, 2014).

As basic information concerning water requirements has started to accrue in the southern hemisphere, more sophisticated approaches are now being examined. For example, it has been suggested from studies in New Zealand and Argentina that plant-based indicators such as fluctuations in trunk diameter and stem water potential have considerable potential for programming deficit irrigation (Greven et al., 2009; Trentacoste et al., 2015; Agüero Alcaras et al., 2016). In this regard, oil yield was only slightly reduced over three seasons when irrigation was applied below a stem water potential threshold of −2.5 MPa in cv. Arbequina in central-western Argentina (Trentacoste et al., 2015). Thus, this technique appears to be promising for reducing irrigation as competition between sectors for water resources increases. In super-high density olive orchards in Chile, there are also significant advances in determining energy balance components using ground-based measurements and unmanned aerial vehicles that have direct applications for irrigation management (López-Olivari et al., 2016; Ortega-Farías et al., 2016).

To summarize this section, the differences in rainfall distribution between some southern hemisphere sites and the Mediterranean Basin have provided new knowledge about water requirements in olive trees. Unlike the Mediterranean, little rainfall during the winter and early spring occurs in many of the main olive growing areas in Argentina and Australia. Assessments of water requirements from flower differentiation to early fruit growth indicate that irrigation during the pre-flowering—flowering period is essential to enhance reproductive performance and oil yields in areas with a dry winter-spring season.

### Oil concentration and composition

The various stages of olive fruit growth and oil synthesis occur over a prolonged total period of 5–6 months (Figure 1). Under the environmental conditions in the Mediterranean Basin, most oil accumulation coincides with the late summer and fall months when temperatures are decreasing from maximum summer values. In contrast, onset of the olive oil biogenesis period takes place somewhat earlier in southern hemisphere growing regions located at relatively low latitudes, and most of the oil accumulation occurs during the summer when temperatures are higher. For this reason, this section will explore the potential importance of temperature on oil concentration and composition in non-Mediterranean climate growing regions.

In the Mediterranean Basin, the dynamics of oil accumulation are considered to have sigmoidal-type curves, irrespective of cultivar, although the rates and duration of the oil synthesis period may vary for a given cultivar according to local environmental conditions (Allalout et al., 2011; Camposeo et al., 2013). A sigmoidal, S-shaped curve includes little increase in oil concentration early in fruit growth before pit hardening, followed by an extended linear period of oil concentration increase, and then a leveling off of oil concentration late in the season. Consistent with this pattern, recent studies in Argentina focused on modeling fruit oil concentration (%) in a number of cultivars from the beginning of pit hardening until harvest described bi-linear relationships where oil concentration increases linearly from pit hardening until it reaches a threshold value, above which oil concentration does not increase further (Trentacoste et al., 2012; Rondanini et al., 2014). In a fairly similar manner, oil synthesis in the local cv. Arauco was low until pit hardening and then followed a saturation curve, first increasing almost linearly, and then appearing to reach a plateau 170 days after full flowering (Bodoira et al., 2015). These results tend to indicate that the overall, basic pattern of oil accumulation is not altered by the climatic conditions in the southern hemisphere.

In contrast, the amount of oil accumulated does seem to be greatly affected by high temperatures. Based on correlative field studies using data from different cultivars, years, and locations in the warm desert region of NW Argentina, Rondanini et al. (2014) found that final fruit oil concentration on a dry weight basis decreased over a mean temperature range of 23°-27°C. Additionally, a comparison of oil concentration values reported from cooler central-western Argentina (45.5–57.4%; 33°S latitude; Trentacoste et al., 2012) with values from NW Argentina (36.5–48.5%; 28–29°S; Rondanini et al., 2014) shows that final oil concentrations were lower in the warm NW region. While such apparent differences are fairly clear when considering new crop environments over significant latitudinal gradients, decreases in oil concentration have been reported even over short geographical distances within the Mediterranean Basin. In this regard, final oil concentrations averaged 18% on a fresh weight basis for the cooler coastal plain and only 14% for a warmer interior valley in Israel (Lavee et al., 2012). Maximum daily temperatures were about 5°C greater during oil accumulation at the interior valley site.

An important approach to directly evaluate the fruit oil concentration response to high temperature has been carried out by heating or cooling fruiting branches (cv. Arauco) in transparent plastic chambers under field conditions in NW Argentina (García-Inza et al., 2014). After 4 months of treatment, this study found a negative linear relationship between oil concentration and mean daily temperature during the oil synthesis period with oil concentration decreasing 1.1% per °C across the range of average seasonal temperatures tested (16–32°C). When temperature was manipulated for shorter periods (i.e., 1 month), temperatures 7°C above ambient resulted in a negative effect on oil concentration even at final harvest, particularly when the exposure to high temperature took place at the beginning of oil accumulation. Such controlled, experimental results provide further evidence that oil concentration is strongly influenced by temperature.

To better understand oil concentration responses to temperature, the influence of temperature on the duration of the fruit-oil filling period and on the rate of oil accumulation needs to be considered. In central-western Argentina, data from several cultivars indicated that the fruit oil-filling period was shortened by about 40 days with increasing maximum daily temperature and solar radiation (Trentacoste et al., 2012). On the other hand, fruit oil concentration was linearly related to the rate of oil accumulation in NW Argentina, but not to the duration of the oil-filling period (Rondanini et al., 2014). Thus, further studies are needed to assess the underlying mechanisms of temperature on oil accumulation.

Regarding olive oil composition, increasing evidence shows that some European olive cultivars grown in many regions of South America and Australia produce oils with different fatty acid compositions compared with those obtained from the same cultivars in their original Mediterranean Basin growing areas (Torres and Maestri, 2006; Ceci and Carelli, 2007; Torres et al., 2009; Mailer et al., 2010; Rondanini et al., 2011, 2014; Bodoira et al., 2016). In some cases, the percentages of fatty acids such as oleic acid do not even meet current International Oil Olive Council (IOOC) trade standards. This occurs despite proper agronomic practices, harvesting, and fruit processing standards.

While genotype is considered to be the major source of variability for VOO fatty acid composition (Ripa et al., 2008), the effect of environment and the genotype x environment interaction may also be significant. Many Spanish (“Arbequina,” “Manzanilla”) and Italian (“Frantoio,” “Coratina”) cultivars show consistently lower oleic acid content and higher palmitic and linoleic acid contents when grown in Argentina vs. the Mediterranean (Ceci and Carelli, 2007; Torres et al., 2009; Rondanini et al., 2011, 2014). In contrast, tree cluster analysis indicated that the fatty acid composition was fairly similar in “Picual” when grown in Argentina or in the Mediterranean region (Rondanini et al., 2011). Additionally, oleic acid (%) appears to decrease to a much greater degree in some cultivars such as “Arbequina” than in others including “Coratina.”

The cv. Arbequina was first introduced to Argentina from Spain about 70 years ago. A study by Torres et al. (2009) using AFLP DNA markers has showed high genetic homogeneity in this cultivar in central Argentina compared to its original Spanish growing region. In central Argentina, oleic acid content in “Arbequina” VOOs is 10–15% lower than in Spanish oils (Table 2), but this difference is unlikely to be explained by a founder effect associated with a relatively low number of “Arbequina” individuals being introduced originally to Argentina. Much newer “Arbequina” orchards established in NW Argentina, which originated from cuttings introduced from Europe in the 1990s, also produce oils with much lower oleic acid contents than oils from Spain.

**Table 2:** Fatty acid composition of virgin olive oils from cv. Arbequina cultivated at different growing areas in Spain (Tous et al., 1997; Pardo et al., 2007), Argentina (Ceci and Carelli, 2007; Torres et al., 2009; Rondanini et al., 2011), and Australia (Mailer et al., 2010).

**Parameters**	**Spain (37°-42° N)^a^**	**North-western Argentina (27°-29°S)^b^**	**Central Argentina (30° 5'S)^b^**	**Central-western Argentina (31°-33°S) ^b^**	**Australia, including Tasmania (29°-42°S)^a^**
Maturity index	Veraison—ripe	3.1 ± 0.6	3.4 ± 0.3	4.5	Veraison—ripe
**FATTY ACIDS (%)**					
Palmitic	11.3–13.9	19.2 ± 1.3	17.5 ± 1.4	15.8 ± 1.2	10.4–19.7
Palmitoleic	1.1–1.2	3.3 ± 0.7	2.5 ± 0.3	1.6 ± 0.3	0.8–3.5
Stearic	1.7–2.4	1.6 ± 0.1	1.4 ± 0.4	1.9 ± 0.2	1.2–1.9
Oleic	69.8–74.6	51.8 ± 4.1	61.3 ± 3.9	63.3 ± 3.1	54.5–81.0
Linoleic	8.3–11.4	21.9 ± 2.8	16.0 ± 3.7	15.5 ± 2.3	4.4–19.4
Linolenic	0.5–0.6	1.0 ± 0.1	0.8 ± 0.1	0.6 ± 0.2	0.6–0.7
MUFAs/PUFAs^c^	5.5–8.4	2.4 ± 0.5	3.9 ± 0.3	4.1 ± 0.4	3.0–8.0

a *Data from Spain and Australia are presented as a range of mean values*.

b *Data from each olive growing region in Argentina were averaged and reported as mean values (± standard deviation)*.

c *MUFAs, monounsaturated fatty acids; PUFAs, polyunsaturated fatty acids*.

Recently, some studies have evaluated the dynamics of fatty acid accumulation during fruit ontogeny in olive cultivars growing in several environments in Argentina (Rondanini et al., 2014; Bodoira et al., 2015, 2016). In all cultivars tested, the oleic acid content had similar maximum values (about 70% of the total fatty acid content) at early fruit growth stages and then it generally decreased, albeit at different times and rates depending on both the olive cultivar and the environment. This could indicate that the enzymatic activities of fatty acid desaturation metabolism are influenced by genotype x environment interactions. To assess this possibility, enzymatic studies are currently being conducted using fruit collected over a wide latitudinal gradient in western Argentina to obtain relationships between enzymatic activities and the levels of oleic and linoleic acids in two cultivars (“Arbequina” and “Coratina”) showing different fatty acid profiles.

Correlation studies of olive oils from the warm valleys of NW Argentina suggest that temperature during the oil synthesis period could be the main environmental factor affecting fatty acid composition of VOOs. In this region, negative relationships between oleic acid concentrations at final harvest and seasonal mean temperatures during oil synthesis have been found for the cv. Arbequina (Rondanini et al., 2011). When the dynamics of fatty acid accumulation were modeled as a function of thermal time, oils from “Arbequina” showed a significant reduction in oleic acid content with thermal time (approximately 0.8% per 100°Cd), resulting in concentrations of less than 50% at final harvest, which coincided with a thermal time of about 3,500°Cd (Rondanini et al., 2014).

More direct evidence of the response of oil fatty acid composition to temperature was found by enclosing fruiting branches in transparent plastic chambers during the fruit filling period for 4 months under field conditions (García-Inza et al., 2014). These authors found that fruits (cv. Arauco) developed under temperatures 5 or 10°C warmer than the seasonal mean ambient temperature (20.6°C) produced oils with lower oleic acid contents. Across the whole range of temperature explored, the oleic acid concentration decreased linearly 0.7% per °C, while palmitic, linoleic and linolenic acids percentages increased with increasing temperature. Interestingly, further study also found that oleic acid content in oils extracted from the seed and the mesocarp showed opposite responses to ambient temperature (García-Inza et al., 2016). In other words, oleic acid content increased with temperature in the seed, but it decreased in the mesocarp. The increase of oleic acid in the seed of olive fruit is consistent with the response observed in oil-seed crops, such as sunflower and soybean (Rondanini et al., 2003; Zuil et al., 2012). Detailed biochemical and molecular research is needed to understand why oleic acid content in the mesocarp decreases with temperature under field conditions. The response of fatty acid composition to temperature has not been a subject of major concern in the Mediterranean Basin. However, it may become of interest with global warming. At the very least, this issue presents a drawback for commercial olive oil production in areas that already have high temperatures.

Similar to Argentina, the wide variations in Australian olive crop environments sometimes result in oils with chemical and sensory attributes being more variable than those observed in oils produced in Mediterranean countries (Ayton et al., 2007; Mailer et al., 2010). Particularly, the fatty acid composition of “Arbequina” is notably influenced by the environmental conditions of the different Australian growing regions (Table 2). The oleic acid content of “Arbequina” VOOs is markedly lower in the northern warmer areas from New South Wales and Queensland (54.5% in average) than in the southern cooler region (81% in VOOs from Tasmania). Variations in oleic acid content in other olive cultivars grown in Australia are also significant and follow the same tendency, i.e., decreased oleic acid content in oils from warmer climates (Mailer et al., 2010). The concentrations of the different fatty acids generally fall within the IOOC acceptable limits, but this is not always the case.

Differences in fatty acid composition attributable to geographic variations can be also found in “Arbequina” growing in Chile where expansion has led to olive oil production in regions varying widely in latitude from 18°S (Azapa Valley) to 36°S (Central Valley). Although there is no evidence of a direct effect of temperature on composition of Chilean “Arbequina” olive oils, Portilla et al. (2014) reported lower oleic acid contents in oils from the most northern latitudes compared with those from the most southern ones (57 and 76% in average, respectively). Interestingly, Brazil and Uruguay are beginning to cultivate olives and to produce VOOs. Brazilian olive production is carried out mainly in the State of Minas Gerais in southeastern Brazil at subtropical latitudes (22–23°S), in an area where the mean annual temperature is about 19°C and fluctuates between 12°C (minimum) and 26°C (maximum), with mean annual rainfall being approximately 1,300 mm. Under these mild temperature conditions, the oleic acid content of olive oils from 11 cultivars with different origins was found to be in the range 70.8–84.3%, and concentrations of all individual fatty acids were within the IOOC standards for VOOs (Ballus et al., 2014).

Overall, this section indicates that olive oil from regions with warmer temperatures often has lower fruit oil concentration (%) and oleic acid content than from regions with more moderate temperatures. In addition, the reductions in oleic acid appear to be cultivar-specific. This suggests that a genotype x environment interaction is likely important in olive oil quality responses to temperature. Attempts to modify oleic acid content with agricultural practices such as irrigation management have so far been unsuccessful (e.g., Berenguer et al., 2006; Vita Serman et al., 2011; Caruso et al., 2014). Only very subtle responses to irrigation level have been observed. This suggests that cultivar selection and potentially breeding will be of significant importance in obtaining olive oil with high oleic acid content in warm areas.

## Conclusions and further research

Increasing global demand for olive oil has expanded olive cultivation to new growing areas in the southern hemisphere. These new crop environments often do not have typical Mediterranean climates, and some of them are in the subtropics where the response of the crop is relatively unknown. Based on the results of recently published studies, this review has highlighted: (1) the occurrence of insufficient chilling hours for flowering during winter dormancy in some high chilling requirement cultivars, such as “Frantoio” and “Leccino” in specific areas; (2) the lack of winter and spring rainfall in parts of Argentina and Australia illustrate the importance of rainfall distribution and indicate that some amount of irrigation is likely needed throughout the entire year to avoid declines in oil yield in some areas; and (3) reductions in oil concentration and oleic acid content in warm areas emphasize what may be expected for cooler regions such as the Mediterranean with global warming.

With respect to selecting specific cultivars for new southern hemisphere environments, the cv. Arbequina, which is the most common cultivar worldwide in modern super-high density orchards, has been shown to flower consistently even in warm subtropical regions, but its oil concentration and oleic acid content are often much lower than when grown in the Mediterranean. Other cultivars have also been shown to have positive and negative attributes in these new environments. Nevertheless, cultivar-specific simulation models are recommended as approximate tools to predict whether individual cultivars will likely flower in proposed new growing areas.

Temperature has emerged as a key variable considering the geographic variability found in the southern hemisphere. A critical aspect of future research may be the response of olive trees to temperature from the biochemical-molecular level to the whole-plant level. It will be important to take into account the considerable genetic variability in olive trees and the apparent genotype x environment interactions that exist for some aspects of olive quality. Thus, the use of many cultivars in studies would be desirable when practical. Lastly, basic studies are not yet available for many growing regions in the southern hemisphere. Such information would enhance our overall understanding of olive cultivation, and reduce the necessity to extrapolate from only a few regions.

## Author contributions

MT, PP, PS, MR, GG, and DM contributed substantially to the conception and design of the review; MT, PP, PS, MR, and DM drafted the text; MT, PP, PS, MR, GG, AM, RB, CC, and DM approved the version to be published; MT, PP, PS, MR, and DM agreed to be accountable for all aspects of the work.

### Conflict of interest statement

The authors declare that the research was conducted in the absence of any commercial or financial relationships that could be construed as a potential conflict of interest.
